# TMPRSS2 in microbial interactions: Insights from HKU1 and TcsH

**DOI:** 10.1371/journal.ppat.1012677

**Published:** 2024-11-20

**Authors:** Zhengyang Pan, Daoqun Li, Leiliang Zhang

**Affiliations:** 1 Department of Clinical Laboratory Medicine, The First Affiliated Hospital of Shandong First Medical University & Shandong Provincial Qianfoshan Hospital, Jinan, Shandong, China; 2 Department of Pathogen Biology, School of Clinical and Basic Medical Sciences, Shandong First Medical University & Shandong Academy of Medical Sciences, Jinan, Shandong, China; University of Iowa, UNITED STATES OF AMERICA

## Abstract

Transmembrane Serine Protease 2 (TMPRSS2), known primarily for its role as a protease, has emerged as a critical receptor for microbial agents such as human coronavirus HKU1 and exotoxin TcsH. HKU1 utilizes both sialoglycan and TMPRSS2 for cellular entry, where sialoglycan primes the spike protein for TMPRSS2 binding. TMPRSS2 undergoes autocleavage to enhance its affinity for the HKU1 spike, facilitating viral membrane fusion postcleavage. Interestingly, TMPRSS2’s catalytic function is dispensable for both HKU1 and TcsH interactions, suggesting alternative roles in pathogenesis. Structural insights highlight potential therapeutic targets against viral infections and cancers, leveraging TMPRSS2 interactions for drug development. Understanding the interplay between TMPRSS2 and microbes opens new avenues for targeting TMPRSS2 in developing treatments for infections.

## 1. Introduction

Transmembrane Serine Protease 2 (TMPRSS2), a member of the type II transmembrane serine protease family (TTSP), plays a crucial role in degrading and remodeling the extracellular matrix near cells, as well as activating membrane proteins essential for maintaining epithelial homeostasis [1]. It was initially identified during the development of the transcription map for human chromosome 21 [2]. TMPRSS2 is present in various epithelial tissues, including the prostate epithelium, kidney tubules, upper airway epithelium, alveoli, colonic epithelium, bile ducts, and ovaries [3]. Under normal conditions, TMPRSS2 facilitates the proteolytic activation of prostate-specific antigen and regulates epithelial sodium channel activity within the prostate gland in vitro [4]. Additionally, respiratory viruses such as SARS-CoV-2, SARS-CoV, and influenza A viruses rely on TMPRSS2 to proteolytically activate their envelope glycoproteins, facilitating fusion between viral and cellular membranes [1,5].

Interestingly, TMPRSS2’s involvement in microbial interactions extends beyond its protease function. In 2022, Li and colleagues [6] identified TMPRSS2 as a receptor for hemorrhagic toxin (TcsH), a potent exotoxin produced by the gram-positive anaerobic bacterium *Paeniclostridium sordellii* (formerly *Clostridium sordellii*). Shortly after, Saunders and colleagues [7] discovered TMPRSS2 as the cellular receptor for human coronavirus HKU1 in 2023. In March 2024, Zhou and colleagues [8] elucidated the molecular mechanism of TMPRSS2 recognition by TcsH. Recently, 5 articles published by Fernández and colleagues [9], McCallum and colleagues [10], Wang and colleagues [11], Xia and colleagues [12], and Gao and colleagues [13] provided detailed insights into TMPRSS2’s role as the receptor for HCoV-HKU1.

## 2. TMPRSS2 is required for HKU1 entry

HKU1 was first identified from a patient with pneumonia in Hong Kong, in January 2005 [14]. Common symptoms following HKU1 infection include fever, cough, rhinorrhea, and wheezing. While HKU1 primarily affects the upper respiratory tract, it can lead to severe illness such as pneumonia in young children, the elderly, and immunocompromised individuals. There are 3 genotypes of HKU1: genotype A, genotype B, and genotype C, which is a recombinant of genotypes A and B [7]. The receptor-binding domain (RBD) of the HKU1C spike is nearly identical to that of HKU1B, exhibiting a sequence identity of up to 98%. In contrast, the sequence identity between HKU1A and HKU1B decreases to 74% [12].

**Fig 1:**
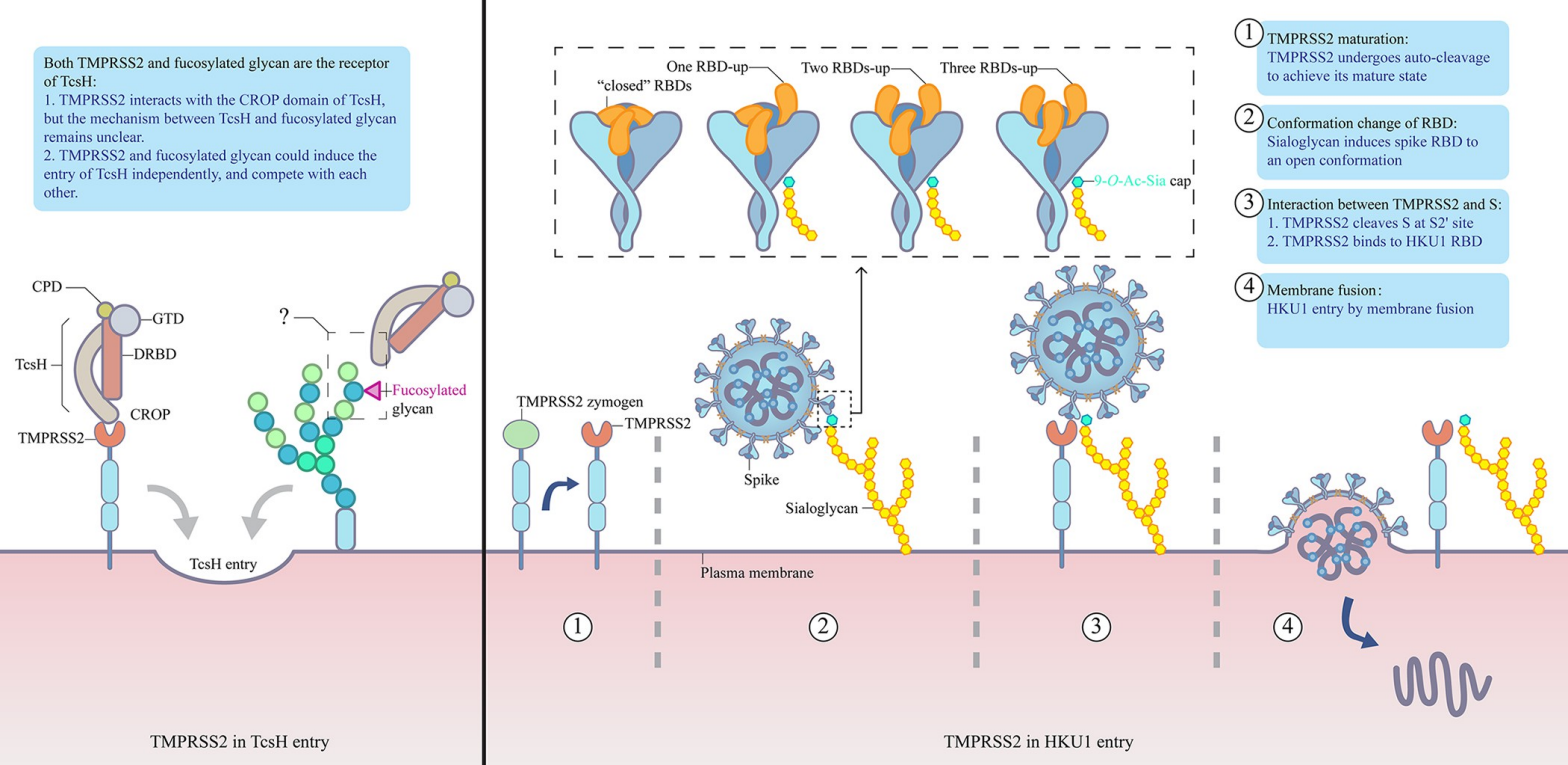
Diagram illustrating the role of TMPRSS2 in the cellular entry of TcsH and HKU1. For TcsH: Both TMPRSS2 and fucosylated glycans act as receptors for TcsH, facilitating its entry. TcsH utilizes its CROP domain to bind with TMPRSS2. For HKU1: TMPRSS2, initially synthesized as an inactive zymogen on the plasma membrane, undergoes autocleavage for maturation, thereby enhancing its binding affinity to the HKU1 spike. Sialoglycan functions as the glycan receptor for HKU1, alleviating steric hindrance upon binding and promoting an “open” conformation of the spike’s RBD, which facilitates spike binding. TMPRSS2 serves as the protein receptor that facilitates HKU1 entry into host cells. In addition to its role as a receptor, TMPRSS2 catalytically cleaves the spike at S2’ site, preparing it for fusion.

TMPRSS2 plays 2 key roles in HKU1 pathogenesis: (1) it acts as a host receptor by binding to the HKU1 spike RBD in the S1 subunit; and (2) it cleaves the HKU1 spike at S2’ site, priming it for membrane fusion (Fig 1). Although the HKU1 RBD binds at the edge of the TMPRSS2 catalytic sites without directly interacting with its crucial catalytic residues, this binding sterically hinders TMPRSS2 substrates and impairs its catalytic activity [9–11]. Moreover, TMPRSS2’s receptor function does not depend on its catalytic activity, as other proteases may substitute for its protease function [7]. Thus, TMPRSS2-mediated HKU1 entry and spike cleavage are separate processes. The coordination between TMPRSS2’s receptor and protease functions is yet to be fully understood. Typically, TMPRSS2 with catalytic activity cleaves the HKU1 spike and binds to it, facilitating viral entry through membrane fusion. For HKU1A pseudovirus entry, inactive TMPRSS2 can be compensated by cathepsin, suggesting that HKU1A might use internalization for entry and subsequent processing in the endosomal compartment [7]. However, the mechanism for HKU1B with inactive TMPRSS2 remains unclear.

The interactions between TMPRSS2 and HKU1 (A and B genotypes) primarily consist of a hydrophobic surface near the catalytic sites of the serine peptidase (SP) domain of TMPRSS2 and a groove that spans residues 503–533, formed by the apex of the HKU1 RBD [13]. The interaction landscape between TMPRSS2 and HKU1C can be divided into 3 distinct interfaces [11]. Main interactions involve hydrophobic interactions and a series of hydrogen bonds [11]. The binding modes of TMPRSS2 across all 3 genotypes are nearly identical. Specifically, for HKU1C, the important residues are Y414, Y469, and V415 from TMPRSS2, and Y528, P522, and L521 from the spike protein in interface I; V415 and R470 from TMPRSS2, and R517, D505, and D507 from the spike protein in interface II; and Y416, K342, W461, and L419 from TMPRSS2, along with T509, L510, and W515 from the spike protein in interface III. In the case of HKU1A interactions, only 3 residues differ: D505E, D507T, and T509V in the spike protein. The variation of HKU1 RBD from D507 to T507 may disrupt a salt bridge between HKU1 RBD and TMPRSS2, which could explain why the binding affinity of HKU1A RBD toward TMPRSS2 is lower than that of HKU1B RBD [13]. A comparison of the HKU1A/B RBD structures bound to TMPRSS2 reveals that most of the key interaction residues are nearly superimposable. Therefore, the difference in binding affinity between HKU1A and HKU1B may be attributed to variations in the side chains [13].

## 3. TMPRSS2 undergoes autocleavage, enhancing its binding affinity in interactions with HKU1

TMPRSS2 is initially synthesized as an inactive zymogen and becomes active through autocleavage (Fig 1) [9]. The cleaved form of TMPRSS2 demonstrates a significantly higher affinity for the RBD of the HKU1 spike compared to its uncleaved precursor [9]. The enzyme features 8 surface loops on its top surface, including LA-E and L1-3, which determine its protease substrate specificity [15]. Cleavage-induced conformational changes particularly affect loops L1 and L2 [9]. In vitro studies indicate that the binding affinity of cleaved TMPRSS2 for the HKU1 spike RBD is approximately 5 times greater than that of the zymogen form [9]. The cleavage process notably alters the conformation of residues G432 (in L1) and S463 (in L2), with G432 moving away from the RBD and S463 extending to form a hydrogen bond with the RBD postcleavage [9]. This conformational shift likely accounts for the increased binding affinity of the mature TMPRSS2.

## 4. Sialoglycan-induced RBD opening is crucial for TMPRSS2-HKU1 interactions

Pronker and colleagues [16] established that 9-*O*-acetylated-sialic acid (9-*O*-Ac-Sia) are the primary receptor of HKU1 and sialoglycan binding could induce the opening of HKU1 spike trimer. Sialoglycan binds at the N terminal subdomain of S1 subunit priming for the conformational change of a neighborhood subdomain of S1 [16]. This active state is short but stable enough to be observed with the spike remaining closed [16]. The RBDs become exposed in a 1-RBD-up or fully 3-RBDs-up conformation priming for binding with the protein receptor (Fig 1) [16]. After TMPRSS2 was identified as the protein receptor of HKU1, in the research carried out by Wang and colleagues [11], a 2-RBDs-up conformation was observed between the 1-RBD-up and 3-RBDs-up conformation (Fig 1). Normally, HKU1 C spike trimers predominantly (approximately 58%) adopt a “closed” conformation where all 3 RBDs point downward [11]. From a top-down perspective, the RBDs in each spike protomer jut out, wedging themselves between the NTD and CTD of a neighboring protomer, creating a woven-like appearance [11]. However, they also found that the trimer could establish a natural 1-RBD-up or 2-RBDs-up conformation in a not-activated state without binding any sialoglycan [11]. More RBDs open means a higher possibility of binding with TMPRSS2. The ability of different genotypes HKU1 spikes to adopt the open conformation spontaneously or after the activation of sialoglycan might explain the difference in some of their pathogenesis.

## 5. HKU1 evolution and its host tropism

While ACE2 binding represents an ancestral trait of sarbecoviruses, the evolutionary significance of TMPRSS2 binding in coronaviruses remains under investigation. TMPRSS2 binding affinity may have been a newly acquired property in HKU1 evolution, contrasting with the ancestral state observed in sarbecoviruses [7]. The HKU1 spike’s receptor-binding motif (RBM) is shielded by a neighboring glycan at position N355, limiting accessibility for antibodies and facilitating immune evasion [10]. The RBD opens only after binding with the glycan receptor sialoglycan, preventing premature exposure to neutralizing antibodies. This coordinated mechanism between RBM shielding and sialoglycan-induced opening reflects an evolutionary balance.

Among TMPRSS2 orthologs, the binding site in HKU1 is most conserved in mammals, followed by reptiles and birds [10]. Certain mutations in human TMPRSS2, such as Y414S/L/R, D417N, L419A, S463T/Y/F, Y469N, and R470K, significantly reduce HKU1 RBD binding [10]. TMPRSS2 residues critical for HKU1 binding, such as D417 and Y469, vary across species, influencing host tropism [9]. Species with TMPRSS2 variants containing these residues, such as camels and alpacas, may serve as potential HKU1 hosts [11]. Some human TMPRSS2 mutations, like D417N and Y469N, occasionally occur alongside Y414 mutations but do not always prevent pseudovirus infection, indicating other contributing factors [10]. These observations help define HKU1’s host specificity. Additionally, nonconservative mutations in the human TMPRSS protease subfamily may explain HKU1’s preference for TMPRSS2 over other members like TMPRSS3, TMPRSS4, or TMPRSS13 [7,10].

## 6. TcsH uses TMPRSS2 and fucosylated glycans as host receptors

*P*. *sordellii* can infect both humans and animals, often leading to severe obstetric infections with a near 100% mortality rate following natural delivery or spontaneous abortion [17]. *P*. *sordellii*’s primary virulence factors are the exotoxins TcsL and TcsH [18]. TcsH binds to both TMPRSS2 and fucosylated glycans, and these receptors can independently facilitate TcsH entry. There seems to be competition between TMPRSS2 and fucosylated glycans for TcsH binding [6].

TcsH is composed of 4 domains: a C-terminal combined repetitive oligopeptides (CROPs) domain, a combined transmembrane delivery and receptor-binding domain (DRBD), a cysteine protease domain (CPD), and a glycosyltransferase domain (GTD) [19] In the TMPRSS2-TcsH complex, TcsH assumes a “closed” conformation with its CROP domain bending downward and the DRBD domain aligned under neutral pH conditions [8] (Fig 1). The interaction between TMPRSS2 and TcsH involves TMPRSS2’s SP domain and TcsH’s CROP domain, with TMPRSS2’s surface loops (LA, LB, LE, L2, and L3) engaging the TcsH CROP domain in a gripping manner [8]. Similar to HKU1, TcsH entry does not rely on TMPRSS2’s proteolytic activity [6]. Binding of TcsH may inhibit TMPRSS2’s catalytic activity by occupying its catalytic pocket [8]. Both TMPRSS2 and TcsH’s CROP domain are crucial for TcsH-related damage in mouse models, with mutated TcsH that binds TMPRSS2 less effectively causing reduced epithelial damage in mouse colons [8].

**Fig 2:**
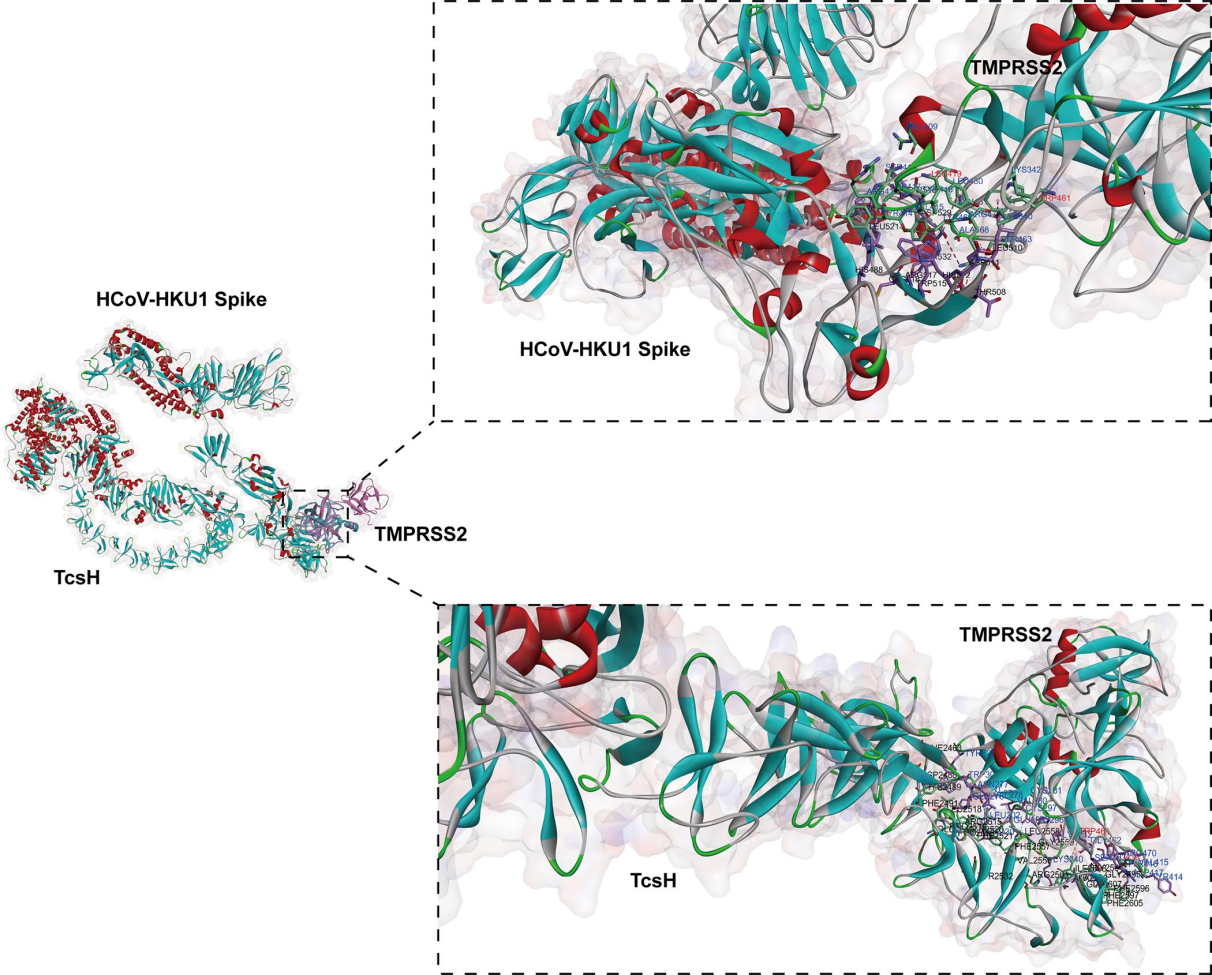
Superimposed structures of the HKU1A spike–TMPRSS2 interface and the TcsH–TMPRSS2 interface. The left panel displays the binding interfaces of the HKU1A spike and TcsH proteins with TMPRSS2. The right panel shows cartoon representations of the enlarged views of the HCoV-HKU1A spike (top) and TcsH (bottom) at the TMPRSS2 interface. The green area indicates the binding interface of the HKU1A spike on TMPRSS2, involving amino acids 256–491. The purple-red region represents the binding interface of TcsH on TMPRSS2, which includes residues 142–492. The residues L419 and W461 on TMPRSS2, labeled in red, are shared interaction residues for both the HKU1A spike and TcsH. The three-dimensional structures are derived from the structure of HCoV-HKU1A spike in the functionally anchored-3up conformation with 3TMPRSS2 (PDB ID: 8Y7X) and the Cryo-EM structure of the TcsH–TMPRSS2 complex (PDB ID: 8JHZ), generated using Discovery Studio 2016 Client and PyMOL 2.3.

Both TcsH and HKU1 bind to the SP domain of TMPRSS2 [8,13]. The interface for TcsH–TMPRSS2 interactions is approximately 150% larger than that of HKU1B–TMPRSS2, with buried surface areas of about 1,130 Å^2^ and 800 Å^2^, respectively (Fig 2) [8,9]. The residues occupied by HKU1A on TMPRSS2 range from 256 to 491 [11]. In contrast, TcsH occupies residues 142–492 on TMPRSS2, fully encompassing those occupied by HKU1 [8]. Both HKU1 and TcsH interact with L419 and W461 on TMPRSS2 (Fig 2) [8,11]. Given the high binding affinity between TcsH and TMPRSS2 (Kd of 0.13 nM) and the shared interacting region on TMPRSS2 with HKU1, the structure of TcsH could provide valuable insights for treating HKU1 infections [6,8]. Additionally, since TMPRSS2 activity is crucial for prostate tumor cells, this structure and its high affinity also offer potential implications for cancer treatment [8].

## 7. Conclusions

This Pearl summarizes TMPRSS2’s multifaceted roles beyond its main protease function, particularly in its interactions with TcsH and HKU1. TMPRSS2 activates through autocleavage, enhancing its affinity for the HKU1 spike. Interestingly, TMPRSS2’s protease activity is not necessary for its interactions with TcsH and HKU1; rather, these interactions inhibit its enzymatic function. Structural analyses of TMPRSS2’s engagement with these microbes suggest potential therapeutic avenues, such as developing antibodies and drugs. Moreover, understanding these interactions offers insights into viral tropism and cross-species transmission, aiding in predicting outbreaks and creating effective vaccines by targeting glycan shielding mechanisms, like removing the N355 glycan. These insights not only deepen our understanding of coronavirus entry but also open avenues for investigating similar mechanisms in other coronaviruses, guiding future research. However, the detailed mechanisms by which TMPRSS2 facilitates the entry of HKU1 and TcsH, as well as how TMPRSS2 coordinates its protease and receptor functions during interactions with HKU1, remain largely unexplored.
